# Making the (Business) Case for Clinical Ethics Support in the UK

**DOI:** 10.1007/s10730-020-09416-6

**Published:** 2020-07-21

**Authors:** L. L. Machin, Mark Wilkinson

**Affiliations:** 1 Faculty of Health and Medicine, Lancaster Medical School, Lancaster University, Lancaster, UK; 2 Royal Lancaster Infirmary, University Hospitals of Morecambe Bay Hospital Foundation Trust, Lancaster, UK

**Keywords:** Business case, Clinical ethics, Clinical ethics support, NHS

## Abstract

This paper provides a series of reflections on making the case to senior leaders for the introduction of clinical ethics support services within a UK hospital Trust at a time when clinical ethics committees are dwindling in the UK. The paper provides key considerations for those building a (business) case for clinical ethics support within hospitals by drawing upon published academic literature, and key reports from governmental and professional bodies. We also include extracts from documents relating to, and annual reports of, existing clinical ethics support within UK hospitals, as well as extracts from our own proposal submitted to the Trust Board. We aim for this paper to support other ethicists and/or health care staff contemplating introducing clinical ethics support into hospitals, to facilitate the process of making the case for clinical ethics support, and to contribute to the key debates in the literature around clinical ethics support. We conclude that there is a real need for investment in clinical ethics in the UK in order to build the evidence base required to support the wider introduction of clinical ethics support into UK hospitals. Furthermore, our perceptions of the purpose of, and perceived needs met through, clinical ethics support needs to shift to one of hospitals investing in their staff. Finally, we raise concerns over the optional nature of clinical ethics support available to practitioners within UK hospitals.

## Introduction

This paper provides a series of reflections of an academic socio-ethicist’s (LM) and a senior clinician’s (MW) experiences of making the case for the introduction of Clinical Ethics Support Services (CESS) within a National Health Service (NHS) Trust. At the time of writing, no CESS was available within the local region for hospital- and community-based practitioners, despite extensive support from healthcare professionals across the Trust for the introduction of CESS. The paper will outline the journey of the academic being approached by the clinician to consider setting up a CESS at the Trust, to being faced with the daunting task of completing a business case, to working collaboratively with clinical colleagues to prepare a proposal to the Trust Executive Board. The reflections will focus on the drivers and agendas underpinning the aspiration to establish a CESS, and the emotions and reactions generated by preparing a proposal for the Trust. The paper will make reference to aspects of the hospital Trust’s business case template and how this was “translated” and “interpreted” when composing a proposal for a CESS. As such, this paper provides key considerations for those building a (business) case for CESS within NHS Trusts. We draw upon published academic literature, and key reports from governmental and professional bodies. We also include extracts from documents relating to, and annual reports of, existing CESS within NHS Trusts, as well as extracts from our own proposal submitted to the Trust Board. We aim for this paper to support other ethicists and/or health care staff contemplating introducing CESS into NHS Trusts, to facilitate the process of making the case for CESS, and to contribute to the key debates in the literature around CESS.

## Clinical Ethics Support in Context

In the late 1970s, clinical ethics emerged as an applied field of biomedical ethics, which focuses on individual cases within clinical practice and the practical consequences, for example the moral and ethical issues that arise out of the clinician-patient/family relationship (Solomon et al. 1991; Sulmasy 2001; Thomasma 1994). Clinical ethics has been described as “a discipline that aims to make ethical decisions more orderly, systematic and rational” and, therefore, “deals with concrete judgements in situations in which action must be taken despite uncertainty” (Pellegrino 1989, p. 702). The aim of CESS is to support healthcare professionals, hospital management, patients, and relatives when confronted with an ethical concern, question or dilemma (Molewijk et al. 2016). CESS have been implemented differently in parts of the world and come in various forms including clinical ethics committees, clinical ethics consultants, rapid reviews, ethics drop-in sessions, moral case deliberation, ethics reflection groups, ethics rounds, and seminars and conferences (Magelssen et al. 2016; Molewijk et al. 2016; UK CEN; Weidema et al. 2016). CESS typically have four functions: (a) consultation on ethical issues relating to clinical cases, (b) participation in development of guidelines for good clinical practice, (c) education, and (d) reflection on ethical issues from the acute clinical setting (Andereck 1992; Førde and Pedersen 2011; Fost and Cranford 1985; Rosner 1985). The education function of CESS has predominately been served by seminars and conferences on clinical-ethical topics open to all hospital employees. Depending on the urgency of the support needed, the full committee, an individual clinical ethicist, or a subset of the full committee typically performs the ethics case consultations. Consultation can involve clarifying the value uncertainty or conflict involved and facilitating consensus. Most CESS offering case consultation assumes a non-directive, facilitative role in consultation (Tarzian et al. 2013).

The development of CESS has tended to start “bottom up” (Slowther et al. 2012) and, therefore, the advent and spread of formal clinical ethics support is often described as a grassroots phenomenon. Clinicians who have requested CESS typically describe the experience as useful (Magelssen et al. 2016) and have tended to seek support for conflict resolution, reassurance about a decision, clarification of issues, new insights on a case, and emotional support (DuVal et al. 2001). However, a range of barriers have been reported that are thought to hinder clinicians engagement with CESS, including the perceptions of consultations as difficult to access, time consuming and/or intimidating (Sokol 2009). Equally, some clinicians are simply unaware of the service, fear that a committee will worsen the situation (Gaudine et al. 2011), or mean losing their autonomy in making decisions over a clinical case (Orlowski et al. 2006). CESS have also been accused of being too remote from the daily activities of healthcare or of being ill-equipped to handle the ethical challenges that are most pressing (Bayley 2006; McCruden and Kuczewski 2006).

CESS began in America and are available internationally, including France, Belgium, Netherlands, Germany, Italy, Switzerland, Croatia, Lithuania, Bulgaria, Israel, and Japan (McGee et al. 2001; Simon 2001; Slowther et al. 2001). CESS in the United Kingdom (UK) provides a unique case when contemplating CESS globally. Typically, CESS in the UK has tended to be organised around clinical ethics committees, seminars, and conferences, whereas Scandinavian countries and the Netherlands have witnessed the rise of moral case deliberation and ethics reflection groups, and American hospitals usually have a clinical ethicist on hand for rapid reviews. Furthermore, CESS in UK is different to other countries such as America, Belgium, and Norway, as it is not a legal requirement for all NHS hospitals to have CESS available (Magelssen et al. 2016). The establishment of CESS in the UK and Europe has been slower than in America (Thornton and Lilford 1995), and until recently, the demand for CESS was growing (Mayor 2005; Slowther et al. 2001; Sokol 2005), with at least 82 NHS hospitals providing CESS to their staff in 2010 (Slowther et al. 2012). However, recent figures on the UK Clinical Ethics Network (UKCEN) membership (Austin 2018) suggest that doctors’ access to CESS has become limited, as the number of registered CESS with UKCEN has decreased. A worrying finding for healthcare professionals facing ethical and moral challenges within a NHS that is under immense financial strain (Morley et al. 2019) and delivering care at a time of great political uncertainty nationally and internationally.

## A Need for Clinical Ethics Support in the UK

With such limited formal support available, healthcare professionals in the UK are reported to most frequently access support from senior colleagues and their peers when dealing with clinical ethical dilemmas. Yet, a report from the Royal College of Physicians showed the limitations with such an approach for those needing support,

…there was an overwhelming impression that consultants did not necessarily have the broad understanding of ethical issues, and in particular the bioethical and legal framework surrounding ethical decisions to act as an entirely safe resource for junior doctors (Royal College of Physicians 2005, p. 34)

Moreover, it has been shown that clinicians often lack ethical sensitivity, failing to identify that a difficult issue or case has an ethical as well as a clinical component (McLean 2009). For some UK Foundation Doctors within the first two years of practice since finishing medical school, this means an absence of role models to serve as moral and practical guides (McDougall and Sokol 2008). These problems could be symptomatic of the lack of ethical and legal training available once doctors qualify. Medical schools cannot be expected to prepare tomorrow’s doctors for all the ethical and legal challenges they will face after graduation (Machin et al. 2020). After all, there is a world of difference between discussing ethical problems in the relative safety of a seminar and acting ethically where the implications suddenly become real (Sokol 2010). At present, the training Foundation Doctors receive has a significant emphasis on technical and clinical competencies (Linklater 2010) and limited content on ethics and law topics (Benson 2014; Levy and Coward, 2010) in order to be better prepared to respond to the ethical uncertainty they face in the first two years after medical school (Machin et al. 2020). However, as others have identified, no members of a healthcare team, irrespective of their seniority, are immune from being faced with ethical dilemmas (Larcher et al. 1997) suggesting a need for ethics and law training and education to be available at all stages of career development (Guillemin et al. 2009).

## Recognising a Need for Clinical Ethics Support in One NHS Trust

Clinical colleagues from a hospital Trust in the North West of England help to facilitate clinical ethics workshops and grade clinical ethics coursework at their local medical school where medical students learn, apply, and critique clinical ethics frameworks to their own and others’ cases. Clinical colleagues appreciate the opportunity to be involved in such activities as they acknowledge that they had not received such in-depth ethical training when they attended medical school compared to current medical students as others have noted elsewhere (Demir and Büken 2016). Senior clinicians also feel the weight of expectations upon them to be able to support junior colleagues when facing ethical and legal challenges, yet are often uncertain how to approach and resolve their own as well as others’ ethical dilemmas.

One particular teaching activity within the medical school are the Clinical Ethics Forums, which introduce the concept of clinical ethics support to social work, medicine, and clinical psychology students (Machin et al. 2019). The Forums are multiprofessional to reflect the membership of real-life clinical ethics committees, and students use common ethical frameworks, such as Four Principles (Beauchamp & Childress 1989), Four Quadrants (Jonsen et al. 1982), and Seedhouse Grid (Seedhouse 2009), to analyse and discuss their own dilemmas they have experienced during their training. For the clinical colleagues that facilitate the student Clinical Ethics Forums, it confirmed the gap in the support available to them when facing ethical uncertainty in their clinical practice, as well as highlighted how support could be available within their Trust.

The recognition of support needed in order to overcome the felt isolation when deciding how to proceed in the face of ethical uncertainty was consolidated after a phone call took place between the authors of the paper (herein referred to as ‘we’, ‘us’, or by our initials) to discuss a particularly troubling case together. During the phone call, MW outlined key aspects of the case, and responded to LM’s prompts and queries in order to get a better sense of where the value conflicts lay. In collaboration, the significant ethical, legal, and professional elements of the case were identified and explored, and possible next steps on how to proceed were considered. The decision to explore clinical ethics support via a clinical ethics committee within the Trust was made, and what follows is a brief overview of the steps taken at the time of writing.

## Establishing the Case for CESS in One NHS Trust

We decided to hold a meeting outside typical work hours with key players within the hospital Trust invited including the Chief Executive Nurse, Legal Advisor, Clinical and Nursing Leads, Research and Development Director, and End of Life Representatives. A Trustee from the UKCEN attended and presented at the meeting, who also had experience of clinical ethics committee work and had acted as a clinical ethicist in the north of England. The purpose of the meeting was to gauge if employees within the Trust also deemed a need for CESS, and if so, to widen the circle of involvement, and expand the core of interested people. The idea being that if staff had learned about the CESS through their respected colleagues, and had contributed to developing the innovation through early discussions, then this may foster future engagement in the CESS if implemented as others have highlighted (Rogers 1983; Wilkerson and Maxwell 1988). We also learned of the importance of co-ownership and co-production for users of future CESS from Scandinavian countries and the Netherlands and their use and implementation of moral case deliberation (de Snoo-Trimp et al. 2017) and, therefore, we explored various formats and functions of a CESS. At the end of the meeting, there was sufficient support and interest in establishing a CESS, and agreement from attendees to take the idea of establishing a clinical ethics committee in the first instance forward to senior leaders within the Trust.

The initial response from the senior leaders was to ask us to complete the hospital Trust’s business case template for the proposed clinical ethics committee. According to hospital Trust’s guidance, a business plan is,

a proposal that seeks authorisation from an appropriate decision-making Body for the allocation of resource associated with change to the business e.g. to deliver a new service… (Trust guidance on completing a business case 2014)

Within the Trust, any business case is reviewed and approved through a hierarchy of senior staff and committees, such as Clinical Director, Finance Committee, Trust Board, and then is either approved, with or without conditions, or rejected or recommended to be resubmitted following clarification of certain points.

There is a long-standing culture of justification surrounding “ethics” in medicine (Stirrat 2015) be that explaining the value of teaching ethics in “packed” medical curriculums (Campbell et al. 2007; Lucassen & Fenwick 2014), or defending the time away from clinical duties for Foundation Doctors to receive training (Kirkham and Baker 2012). Most ethicists are, therefore, experienced in, and prepared to, “market” the worth and value of “ethics” (Papanikitas 2018). Yet, we could not find or access any business cases for existing UK CESS when first starting out, and using the internet search term “business case for ethics” unearthed material with limited content (see UKCEN 2014). Whilst we were inexperienced in writing business plans, and the length of the business plan template was unappealing, the real obstacle lay with the “evidence” required to complete it, which is explained further below.

## The “Evidence” Problem When Completing a Business Case Application for CESS

Few commentators on CESS appear to seriously doubt their importance and utility, but as healthcare has become a field dominated by questions of performance, efficiency, and cost effectiveness (Millstone 2014), ethics support appears to need to demonstrate its value to “justify adequate resourcing” (Royal College of Physicians 2005) and show that it does “not waste resources” (American Society for Bioethics and Humanities 2009). While it seems self evident that CESS in the institutional setting may improve the ethical dimension of patient care and so improve the overall quality of healthcare, evidence in support of this contention is hard to come by (UKCEN 2014). In particular, evidence that considers the efficiency of the ethics consultation through the cost savings that it generates (Bacchetta and Fins 1997; Daly 2000; Heilicser et al. 2000) or the effectiveness of the ethics consultation service through satisfaction studies (Tulsky and Fox 1996) is scarce.

We searched the literature and found bold claims espousing the many benefits of introducing CESS for healthcare organisations, including increasing patient satisfaction (Kaplan et al. 1989; Tierney et al. 2001), and improving employee morale (Bischoff et al. 1999), although it was not always clear if a direct causal relationship existed between these outcomes and the presence of a CESS. Others justified the moderate cost necessary for the running of any CESS by claims of improved efficiency and savings, and improvements in clinical quality and safety (Macdonald and Worthington 2012). For example, effective CESS have been shown to improve quality of care and reduce length of stay and cost. Such claims are supported in the wider business and management literature discussing the importance of developing a strong ethics culture within an organisation in order to improve performance, efficiency and productivity (Verschoor 1999). However, methodological obstacles exist in providing the evidence demanded by the business case application, such as determining how to measure the “value” of the support provided by the CESS (Schildmann et al. 2013), agreeing upon how it is “valued” by the various stakeholders (de Snoo-Trimp et al. 2017), and how meaningful any analysis would be based on the low number of cases supported by the CESS (UKCEN 2014).

Exacerbating our challenge of completing the business case application were articles advising to stay clear from those few studies that attempted to construct the “evidence-base” for CESS, particularly those that refer to the cost implications arising from ethical conflicts (see, for example, Nelson et al. 2008) or the financial considerations for CESS (see, for example, Bacchetta and Fins 1997; Daly 2000; Heilicser et al. 2000). In the literature, we are advised that cost savings are not particularly meaningful in the absence of estimates of the costs of a service and, more importantly, do not capture the “intangible benefits” created by CESS (Mills et al. 2005). Instead, we are encouraged to focus on the *value* achieved through CESS’ contribution to the “overall good of the patient” (Repenshek 2017) and the intellectual capital (ethics knowledge) of CESS that create important, but intangible benefits, such as relieving “moral distress” (Mills et al. 2005), improving communication and relationships between healthcare professionals (de Snoo-Trimp et al. 2017). An additional problem proposed with using cost savings as an outcome is the potential for the loss of trust if clinicians or patients come to perceive cost as the primary objective of introducing CESS into a Trust rather than supporting staff and patients when facing ethical uncertainty (de Snoo-Trimp et al. 2017). For example, the expressed aim of the CESS at Great Ormond Street Hospital (GOSH) “isn’t to avoid costly court battles or to save the NHS money. It’s about making the right decision for the right reasons” (Great Ormond Street Hospital 2017, p. 8). That said, European researchers have pointed out the subtle, but important, distinction between the goals and outcomes of CESS when contemplating any financial benefits of CESS. For them, the drivers for CESS to be established and exist do not need to be linked with financial gains, but it is feasible and acceptable for financial benefits to result from CESS and for them to be acknowledged (Schildmann et al. 2013).

We were uncertain of how next to proceed. We recognised the constraints we were working within—NHS funding crisis, age of austerity (Morley et al. 2019)—and had some sympathy with the need to justify spending on introducing CESS into the Trust. Equally, at the Trust, the main forum for discussion of ethical issues and dilemmas was within individual clinical units, although occasionally ethical aspects of particular cases were discussed at regular hospital clinical presentations, e.g., Grand Rounds, Schwartz Rounds. Such discussions were considered ad hoc, unstructured, and constrained by time. Individual members of multidisciplinary teams tended to discuss issues within their own discipline and often with their peers, rather than with their seniors or juniors. Similar to other hospitals, there was a general view that this was unsatisfactory, as it could lead to tensions within teams and issues unresolved as others have noted elsewhere (Hamric and Wocial 2016). More public discussion of ethical issues away from acute clinical settings was, therefore, considered necessary and perceived as being achieved through introducing CESS that had consultative and educational functions, which other researchers have reported as beneficial (Larcher et al. 1997).

The literature confirmed that healthcare staff need reflective spaces within institutions in which to explore and communicate values and ethical obligations as they undergird goals of patient care (Hamric and Wocial 2016) and the CESS could play a role in creating and designing these spaces, and ensuring they remain “open, accessible and active” (Walker 1993, p. 38). We, therefore, decided to pull out the key themes within the business case application and write a proposal instead that acknowledged them. This enabled us to use the evidence available within the literature, including making the argument to avoid using financial considerations as a deciding factor on whether to introduce CESS or not into the Trust. However, the overwhelming financial focus of a business plan can generate a return on investment mindset for both the author and reader, and in turn the grandeur of claims surrounding the impact and influence of the proposed CESS can escalate. In order to demonstrate the “value” and “contribution” of the proposed CESS in our proposal then, we felt inclined to identify and outline a number of problems that could be resolved via the CESS, which created another problem as described next.

## The “Problem–Solution” Problem When Completing a Business Case Application for CESS

The challenge with “problems” when making a business case for CESS is that the “problems” cannot be seen to return. Instead, they should be eradicated in order to justify and warrant the investment of funding into CESS. In effect, a business case application constructs a “problem–solution” mindset whereby the author lays out a problem that needs resolving and identifies possible solutions. In the case of CESS, the discussion falls into “preventive” ethics. Blake (2000) argues that CESS should be proactive to effect changes, rather than merely reacting to the cases that staff bring. The focus and purpose of CESS is then to influence and inform organisational culture. No longer is it the responsibility of one person or one group, but instead the responsibility of the whole institution that ethical practices are enacted. For example, the Ethics Program at the American hospital, Beth Israel Deaconess Medical Center in Boston, acts as a standing mechanism to influence and maintain a moral culture within the institution that,

helps to shift the influence of those who “do ethics” in the institution to everyone who works here, whether at the bedside, in the laboratory or in the office. The programme enables us to enact our mission statement that ethics is part of everyone’s daily work (Bates et al. 2017, p. 597)

Recent attention has been given in the literature to providing assistance with organisational ethics, whereby the ethical issues involved in areas such as management and resource allocation and quality improvement are worked through (Dorries et al. 2011; McClimans et al. 2012). This development responds to the critics of CESS who claim the crucial ethical issues at stake are general, structural, and organisational (Bayley 2006; McCruden and Kuczewski 2006). In essence, what each individual healthcare professional can and cannot do is arguably influenced by institutional policies and procedures, perceptions of legal liabilities, budgetary constraints, established patterns of communication and the tenor of the organisational culture (Solomon et al. 1991). It also reflects the rise in a “systems” approach, whereby clinical ethics are fully integrated into the institution and wider healthcare apparatus (Fox et al. 2010; MacRae et al. 2008; Singer et al. 2001). CESS assist healthcare professionals in resolving value conflicts, whilst also improving respect for values, such as autonomy and equity, in practices at an organisational level (Dörries et al. 2011). For example, CESS can contribute to the development of patient-centred policies on end of life decisions, resuscitation, or filming surgeries, and ensuring they are sufficiently responsive to patients’ preferences, and respect the dignity of patients (McClimans et al. 2011). CESS are, therefore, portrayed as providing “ethical leadership” by improving the ethical environment and culture of the organisation (Magelssen et al. 2016).

The broader view required from the clinical ethicist when shifting focus to the ethical issues at an institutional level, means that it is possible to become aware of the recurring matters that come to the ethical committees. The cases highlight for clinical ethicists “gaps” or other inadequacies in policies that need addressing, and where research is needed (Mills et al. 2005). Some commentators present these recurring ethical issues within an institution as the characters changing within a specific case, but the basic problem is the same (Dunn et al. 2016). Consequently, the role of CESS moves from merely “reacting” to ethical conflicts as they arise to pre-empting them and responding through a system-wide approach (Dunn et al. 2016). In a systems approach, ethics support moves “upstream” to address systematic and structural elements that produce value conflict rather than remain only at the level of the particulars of the issue or case at hand. This encourages a more proactive and preventative form of ethics support (Fox et al. 2010; MacRae et al. 2008; Nelson et al. 2010). The ethical issues and difficulties are perceived as recurring expressions of problematic systemic structures and processes within and between the micro and macro systems within an organisation. Essentially, the cases for the CESS are considered the “tip of the iceberg” (New South Wales Government Health 2015).

Addressing recurring ethical challenges is important as these can create moral distress for the staff within the organisation. Positioning CESS as playing a role at both individual and institutional levels could be crucial as discussions could lead to development of ethics practice strategies to address and possibly decrease the stressful nature of ethical conflicts (Nelson 2009). Staff may feel at ease regarding a decision related to an ethical problem. Staff may be able to make decisions in the best interests of their patients, have their ideas about patient care considered in the care plan, or may be able to relieve or reduce patients’ pain and suffering (Corley and Minick 2002). The aspiration of creating moral comfort for staff is reflected in the aims of an active American CESS,

to promote a culture in which all BIDMC staff appreciate the importance of the ethical aspects of their work (their decisions, actions, character and morale), and have the support they need to do that work in accordance with BIDMC’s, and their own highest moral standards (Bates et al. 2017, p. 595)

It is important to note within the literature that it is more likely that the *emotions* around the conflict are addressed and hopefully reduced, rather than the conflict itself. Arguably, knowing how to resolve a problem or having the means available to respond to a problem, does not mean that the problem itself ceases to exist, or that the response is flawed if it recurs, or that we will not ever have problems to respond to again in the future. The danger of the problem–solution mindset in a business case then is that the discussion becomes unrealistic, with promises of scandals averted, and conflicts and disagreement with next of kin or patients eradicated, thereby setting up CESS to “fail”. Resisting the temptation to make such promises was challenging when composing the proposal. The Trust itself had received negative publicity, with reports of dysfunctional teams, and poor standard of care within a number of departments (see, for example, Dyer 2019 and Goodwin 2019) and CESS have been implicitly presented by various stakeholders as a response to, and a way to prevent, the failings of the NHS healthcare system equating to poor patient care and safety. We briefly outline the challenges below.

## The Problems with Healthcare “Scandals” and CESS as the Solution

The first problem with the association between the “problem” of poor teams and care practices and the “solution” of CESS was how it might influence how healthcare staff perceived CESS. It needed to be handled delicately in order for the CESS not to be perceived by healthcare staff as a “court of law” that produced “ethical verdicts” on clinical cases and on health professionals’ conduct, which others have claimed elsewhere (Pedersen et al. 2009). Equally, such breaches of ethics in NHS Trusts without oversight and correction are arguably detrimental to a Trust’s reputation as well as financially (Dunn et al. 2016). Hospital leaders have to consider carefully how they want to be perceived outside the organisation, so there may be reputational and branding benefits arising from introducing CESS into Trusts after such high profile scandals, inquiries, and reports. With increasing concerns about the inhumane nature of some modern medicine (Weatherall 1994), CESS could promote reflection on ethical matters (Larcher et al. 1997), and could, therefore, be seen as responding to the needs of staff and patients. In turn, an effective CESS could make it easier to recruit and retain quality staff (Francis 2001).

The second problem with the association of CESS with healthcare scandals was the lack of explicit recognition to the transformative influence of CESS in recent healthcare inquiry reports and recommendations. This is surprising given the specific problems identified in the reports such as dysfunctional team work and a lack of communication between multidisciplinary teams, crippling hierarchies, and difficulty speaking up, professional obligations to a duty of candour—all of which could arguably be addressed through, and fall under the remit of, CESS (Carrese et al. 2012; Talash et al. accepted). At a minimum, members of CESS could role model the behaviours such as humility, respect for others, building positive relations, when facing ethical uncertainty (Carrese et al. 2012; Talash et al. accepted). CESS could also help to establish the conditions for ethical reflection, i.e., trust, role flexibility, and enquiry (Brown 1990). So, on the one hand, whilst we were anxious to avoid the damaging impact upon staff’s engagement within CESS arising from the association of CESS as a response to healthcare scandals, we equally struggled to find explicit references that made the association on our behalf to highlight how CESS could address issues raised previously within the Trust.

We, therefore, had to be creative and look to other sources. The UKCEN referred to the extensive failings observed in UK Trusts such as Mid Staffordshire, Basildon and Thurrock when making the case for CESS in the NHS (UK Clinical Ethics Network 2014). Others have conveyed a similar message (see, for example, Beyleveld et al. 2002) including the Care Quality Commission (CQC) who monitors and inspects NHS Trusts to ensure people receive safe, compassionate and high quality care (Care Quality Commission 2019). The CQC has recognised the important role of CESS in their reports when visiting hospitals where CESS exist, such as Great Ormond Street Hospital (GOSH), “The ethics committee was regularly available and played a key role in considering difficult treatment decisions” (Great Ormond Street Hospital 2017, p. 6). This quote from the CQC is then followed within the GOSH CESS report by a warning, “It is worth remembering that the recent Francis Inquiry Report highlighted what can happen if these issues are not prioritized” (Great Ormond Street Hospital 2017, p. 6).

In order to maximise this implicit association between healthcare scandals and CESS, we considered the ideal time to share our proposal with the hospital Trust Executive Board would be ahead of an upcoming visit from the CQC. However, we wished to avoid staff perceiving the CESS as a mechanism of scrutiny (Førde et al. 2008; Gaudine et al. 2011; Slowther et al. 2001) to instil discipline or a form of punishment and, therefore, opted to share the proposal when it was completed. Following the lead of GOSH, we pitched our proposal as having the potential to positively impact upon the organisational culture through supporting staff, supporting the organisational vision, and meeting the government statement on patient decision making,

The introduction of CESS at [hospital Trust] will engage patients and their relatives in discussions when healthcare professionals make difficult healthcare decisions. Therefore the CESS supports [hospital Trust’s] desire to consistently deliver an excellent experience that exceeds patient and family expectations, as well as meeting the government’s vision for shared decision-making in healthcare: ‘no decision about me, without me’ (Extract from LM and MW Proposal for Clinical Ethics Support Service in NHS Hospital)

We considered it less threatening to market the proposed CESS as a way of providing a reflective space, whereby the experiences of patients and relatives could be acknowledged and considered by healthcare staff, rather than reinforcing the association that can sometimes emerge between CESS and someone acting unethically (Carrese et al. 2012). The inclusion of CESS in the Trust would arguably, therefore, act as an explicit way of promoting shared decision-making, and in turn foster institutional awareness around “no decision about me, without me”. Implicitly, at an individual level, CESS had the potential to inform staff’s attitudes and perspectives, thereby acting as a form of staff development. To achieve this, however, it required a shift in how we perceived CESS, in particular their roles and remits.

## The Need for Shifting Perceptions When Writing a Business Case for CESS

As others have identified, doing clinical ethics needed to mean making ethical reflection an integral component of clinical practice, and an explicit part of the daily life of an organisation, and not just considered in the midst of or in response to a crisis (Rubin and Zoloth 2004; Weidema et al. 2016). Yet, CESS have tended to be discussed around high profile or difficult cases, such as the Charlie Gard or Asha King in the UK (for example, see Austin 2018), which has created a sense that everyday ethics do not warrant CESS. The “ethics of the ordinary” are those situations that are part of everyday practice, that occur frequently, and cause a chronic feeling of moral distress (Corley and Minick 2002). Furthermore, there has been a tendency to focus CESS around medical specialisms, departments, and/or types of decisions or groups of patients, such as paediatrics (Gold et al. 2011; Larcher et al. 1997), or intensive care (Schneiderman et al. 2003). However, ethical dilemmas arise frequently in clinical practice, irrespective of the type of unit or its area of speciality (Larcher et al. 1997). CESS services are typically available within acute-care hospitals and as a result, support for community-based practitioners when facing ethical uncertainty is less commonly implemented and, therefore, can be considered an under-serviced population. Small studies show where CESS have been piloted within community healthcare settings, respondents have overwhelmingly reported the services meeting their needs, typically dealing with everyday ethical issues revolving around patient autonomy, privacy, confidentiality, and consent (Racine and Hayes 2006).

These perceptions of when CESS are warranted may provide alternative explanations for the reported low referral of cases to UK CESS (Bates et al. 2017; Slowther et al. 2012), and the isolation and loneliness staff experience around ethically troubling matters. Reports of moral distress (Lamiani et al. 2017; Oliver 2018), predominately focusing on nurses and beginning to be recognised in doctors, highlight that staff can be uncertain about what is the ethically appropriate action to take, and may keep this uncertainty to themselves and not share their stress because they think they are alone in their uncertainty. Alternatively, staff may raise ethical questions, but feel unsupported in acknowledging their uncertainty and are without an available resource to share and discuss the issue. Equally, staff may know or believe an ethically appropriate course of action to take, but are unable to carry out the action because of an organisational obstacle (Lamiani et al. 2017; Nelson 2009).

Such moral distress has consequences for healthcare organisations as it can negatively influence staff morale, confidence, sense of purpose, integrity, and respect for the organisation (Nelson 2009). It also has implications for the wider healthcare service as the higher the level of moral distress, the greater the likelihood of leaving a position (Hamric and Blackhall 2007; Oliver 2018), which is significant in the UK where we are informed that there are insufficient healthcare professionals to cope with patient demand (British Medical Association 2018; Kinman and Teoh 2018), and challenges exist around staff recruitment and/or retention in certain healthcare specialities (British Medical Association 2017; Chaudhuri et al. 2013). In the UK, the General Medical Council are conducting a review of the wellbeing of medical students and healthcare staff, and attempts are being made to support NHS staff through the provision of reflective spaces (see, e.g., Gannon 2014) as recognition increases of the emotional burden that staff carry in their daily work (see, e.g., Cornwell and Fitzsimons 2017; Kerasidou and Horn 2016;). We are also witnessing the resurgence of Schwartz Rounds (Goodrich 2018) and the introduction of resilience training (Oliver 2018). Yet, some existing CESS describe themselves as providing “a space to give clinicians and families a place to discuss their concerns” (Great Ormond Street Hospital 2017, p. 8), raising questions why staff have not approached CESS within their hospitals more frequently (Whitehead et al. 2009). Previously, underutilisation has been linked to a lack of understanding of the role of CESS (Bates et al. 2017) and how it might help (McLean 2009). Arguably, a mismatch may exist surrounding the perceived role—reflective space—and remit—ethics of the ordinary—of CESS between the providers and (potential) users of CESS. Collaborating with CESS could then become part of delivering everyday care, rather than staff approaching the service as a last resort (Hamric and Wocial 2016).

## Concluding Thoughts

Reflecting on the process of proposing CESS into an NHS Trust has highlighted two overarching points. Firstly, the significance of research when making the case for CESS emerged when faced with the business case application. Whilst drafting the proposal, we realised that the evidence available focusing on the UK context was limited, which might be a consequence of the voluntary nature and the lack of any specific requirement for UK healthcare organisations to have a mechanism for addressing ethical dimensions of the work. Historically, CESS have received little or no administrative support or funding, and members’ time has been given voluntarily meaning that any formal evaluation of CESS has been a lower priority for the sparse resources available (Steinkamp et al. 2007; UK Clinical Ethics Network 2014). By searching for the evidence to fill a business case application, we became aware of the urgent need for up to date, empirical studies that analyse and evaluate the UK CESS experiences, which reflect the realities of facing an ethical dilemma within the NHS environment, and can enable sharing of good practices from current CESS in UK. The empirical research needed should draw on both qualitative and quantitative data that goes beyond simple description and is of interest to a range of stakeholders including ethicists, healthcare professionals, patients, and management (Schildmann et al. 2013). Singer et al. (1990) have described the ways in which empirical research can advance both theory and education in clinical ethics. We cannot underestimate the cultural context of discussions surrounding CESS and healthcare systems generally. In our experience, we were bolstered by the position that a lack of available evidence does not mean abandoning a good idea (New South Wales Government Health 2015). We realised that the CESS proposed could have a research function to it and, therefore, fill some of the gaps in the UK evidence base. For example, we proposed being able to provide CESS that serves both hospital- and community-based practitioners, which would position our proposed CESS as a beacon for ethical, innovative and collaborative practice, and enable research into CESS that crosses practice boundaries.

A second overarching point that emerged when making the case for CESS was the significance of how CESS is perceived by all parties in healthcare. Our experiences highlighted that for some NHS staff, limited support is available when they face ethical uncertainty, and some staff lack prior training on ethics and law to prepare them on how to deal with ethical uncertainty. Introducing CESS into a Trust, therefore, should be perceived as a form of *staff investment*. CESS can fill the current gap of ethics and law training after medical school in the UK. Our reflections have emphasised that clinical ethics training can benefit staff at all stages of their career, and the educational and training arm of CESS can, therefore, be viewed as supporting the continuing professional development of Trust staff (Hamric and Wocial 2016). Our reflections have also highlighted that the perceived remit of CESS in the UK needs to evolve so that CESS can be seen as a source of everyday ethics support, which is causing moral distress, rather than solely for the high profile, and controversial cases, or after a Trust has been caught up in a healthcare “scandal”. It is worth noting that since the global pandemic of COVID-19, the NHS Trust discussed in this paper, along with many others across the country, established a Clinical Ethics Advisory Group. However, whether these Groups continue beyond the pandemic remains to be seen. The pandemic has brought to light the value and need for CESS, but again the services are being utilised for the “extreme” rather than the everyday ethical matters that arise from providing care. There is a real need to (re)educate those potentially engaging with and financially supporting CESS in the UK so that it is perceived as providing opportunities for ethical reflection and a space to share and gain—something achieved in Scandinavian countries and the Netherlands as witnessed by the increasing use of moral case deliberation within healthcare settings (de Snoo-Trimp et al. 2017). At a time when much controversy exists surrounding reflection for UK doctors (Dyer and Cohen 2018; Launer 2018), it is vital that the known benefits of ethical reflection, particularly within a group, are not overshadowed (Hem et al. 2018).

In order to shift our perceptions of CESS, we do need to acknowledge the need for ethics support for everyday challenges, for senior as well as junior staff members, for those working in the community, as well as in the hospital. If viewed in this way, CESS sends the message from the organisation that people matter (Levine 1977). Introducing CESS into a Trust can, therefore, be part of a package of support that meets the emotional and wellbeing needs of all staff. The role of CESS can, therefore, be perceived as building and fostering a community, exploring and developing opportunities for staff to share and talk to each other when facing everyday ethical uncertainty, and support organisations to develop policies and practices that promote ethical decision-making. When viewed in this way, the aims of the CESS can then be to reduce the ethical and emotional isolation reported by others (Oliver 2018) through providing support and training. Others have identified the need for organisations to ensure employee health programs are readily available to staff, so that employees can discuss in a confidential setting emotional issues arising in the work environment (Hem et al. 2018; Nelson 2009). There is no one size fits all, however, and CESS should be viewed as part of a tool kit, that compliments existing, but different, support such as Schwartz Rounds and resilience training where they are available (Gannon 2014; Oliver 2018).

If CESS are perceived as investing in staff through providing continuing professional development and supporting emotional and wellbeing needs, any evaluation of CESS also needs to reflect this alternative perception. Some CESS use the level of engagement from all staff in activities relating to ethics within their clinical ethics program (Bates et al. 2017), whereas others consider “success” as improving the level of ethical reflection among clinicians (Magelssen et al. 2016). When proposing introducing CESS into a Trust, we need to be prepared to reframe the “problem”, e.g., ethics and law training, ethical leadership, everyday ethical uncertainty, chronic low-level moral distress. So, when facing the “problem–solution” mind-set generated by a business case application, our language can shift from an erasure and eradication of a “problem”, and importantly, we can justify the constant and permanent presence of the CESS “solution”. By shifting our perception of the role, function, aims, and remit of CESS, we can better manage our own and other’s expectations surrounding CESS. We can reduce the “hype” around what CESS can deliver and achieve, which is generated when completing a business case application, and ultimately setting ourselves up to fail. Instead, the financial focus shifts to investment in our staff, patients, and healthcare system, through education, training, leadership, development, wellbeing, and support. Whereas business plans demand timescales and deadlines, which set up a sense of finality and completion, the shift in our perceptions of CESS mean that we can see CESS as meeting on-going, lifelong needs. Just as these needs will evolve, so should the format and structure of CESS. Viewed in this way, we should all be greatly concerned at the dwindling number of CESS in the UK (Austin 2018), and question the optional nature surrounding NHS hospitals providing CESS for their staff and patients (Beyleveld et al. 2002; UKCEN 2014). As others have argued elsewhere, there is a need for the NHS to be viewed as a moral body, where its staff are part of a moral community and make moral decisions on a daily basis, and they need support to do this (Austin 2007; UKCEN 2014). Healthcare institutions have “ethical lives and characters just as their individual members do” (Reiser 1994, p. 28), and, therefore, need to make a public commitment to ethical issues—which can be achieved through introducing CESS (Royal College of Physicians 2005; Solomon et al. 1991) —in order to fulfil its duty of care to their staff and patients (UK Clinical Ethics Network 2014).

In this paper, we have included extracts from our proposal submitted to the Board, we have outlined the issues that we considered when developing our proposal as well as the evidence that we found and drew upon when making the case for CESS. We welcome dialogue with others interested in our full proposal and experiences. In the meantime, we offer some key considerations when making the case for CESS in the UK. Firstly, engage senior leaders and colleagues within the Trust early in the discussions around CESS and collaborate with senior leaders and colleagues within the Trust when designing CESS. Secondly, seek external support, advice, and expertise. Approach established clinical ethics committees, involve ethicists at local medical schools, and draw on resources from formal clinical ethics networks. Thirdly, gather support from senior leaders within the Trust to work outside any paperwork expectations such as business case application when making the case. Be prepared to engage with the themes, but present the material in alternative formats that shift the discussion beyond simple economics, and the problem–solution approach. Fourthly, be realistic as to the goals and gains of CESS. Do not be tempted to overhype the impact and influence of CESS in order to secure support from senior leaders and colleagues. Understand and utilise the context that provides the backdrop to the CESS you are attempting to establish and be open and honest about it; for example, state that a lack of research on UK CESS exists, remind readers of the lack of ethics and law training nationally after medical school, and highlight the lack of UK CESS available for healthcare professionals to draw on. Fifthly, be mindful of your own and other’s expectations of CESS. Even when and where CESS exist, conflicts can still escalate, and can require legal intervention. As Somerville (2004) points out, do not be tempted to present CESS as a perfect system. When composing our proposal for introducing CESS into the hospital Trust, we acknowledged some of the criticisms towards CESS within the literature and balanced them with the potential benefits of CESS, alongside the present reality in the Trust for staff and patients faced with ethical uncertainty and little formal support available. Sixthly, keep in mind the readers of your proposal. Others (see Dubov 2015; London 2000) have drawn on Aristotle’s work, *Rhetoric*, in the context of healthcare and communication to highlight the importance and power of persuasion, “people make choices and calculate associate risks not only based on what they think about an option, but also on how they feel about it” (Dubov 2015, p. 500).

Therefore, the emotion and needs of the readers can inform and influence how the proposal is written and how the option of a CESS is presented, without losing the integrity of the proposed CESS. Finally, we encourage others contemplating introducing CESS into their UK hospitals and going through a similar process to share their learnings so that we can build and share resources and materials when making the case for CESS.
